# Urethral metastasis from a sigmoid colon carcinoma: a quite rare case report and review of the literature

**DOI:** 10.1186/1471-2482-14-31

**Published:** 2014-05-21

**Authors:** Shinsuke Kazama, Joji Kitayama, Eiji Sunami, Aya Niimi, Akira Nomiya, Yukio Homma, Toshiaki Watanabe

**Affiliations:** 1 Division of Surgical Oncology, Department of Surgery, Faculty of Medicine, The University of Tokyo, 7-3-1 Hongo, Bunkyo-ku, Tokyo 113-8655, Japan; 2 Department of Urology, Faculty of Medicine, The University of Tokyo, 7-3-1 Hongo, Bunkyo-ku, Tokyo 113-8655, Japan

**Keywords:** Urethral metastasis, Colon cancer, Immunohistochemistry

## Abstract

**Background:**

Urethral metastatic adenocarcinoma is extremely rare. Moreover, only 9 previous cases with metastases from colorectal cancer have been reported to date, and not much information on urethral metastases from colorectum is available so far.

**Case presentation:**

We report our experience in the diagnosis and the management of the case with urethral metastasis from a sigmoid colon cancer. A 68-year-old man, who underwent laparoscopic sigmoidectomy for sigmoid colon carcinoma four years ago, presented gross hematuria with pain. Urethroscopy identified a papillo-nodular tumor 7 mm in diameter in the bulbar urethra. CT-scan imaging revealed the small mass of bulbous portion of urethra and solitary lung metastasis. Histological examination of the tumor obtained by transurethral resection showed moderately differentiated adenocarcinoma, which was diagnosed as a metastasis of a sigmoid colon carcinoma pathologically by morphological examination. Immunohistochemical analysis of the urethral tumor revealed the positive for cytokertin 20 and CDX2, whereas negative for cytokertin 7. These features were consistent with metastatic adenocarcinoma of the sigmoid colon cancer. As the management of this case with urethral and lung metastasis, 6-cycle of chemotherapy with fluorouracil with leucovorin plus oxaliplatin was administered to the patient, and these metastases were disappeared with no recurrence of disease for 34 months.

**Conclusion:**

Urethral metastasis from colorectal cancer is a very rare occurrence. However, in the presence of urinary symptoms, the possibility of the urethral metastasis should be considered.

## Background

Urethral tumors are rare. Most of urethral tumors are primary origins, and Surveillance, Epidemiology and End Results (SEER) study reported that an annual age-adjusted incidence rate of primary urethral tumors was 4.3 per million in males and 1.5 per million in females in the United States [1]. Histologically, squamous cell carcinoma or transitional cell carcinoma is the common types, accounting for about 80% of all cases. Adenocarcinoma from paraurethral glands accounts for 10-20% of urethral primaries [2]. In addition, melanomas and various sarcomas have been reported. On the other hand, urethral metastatic tumor, especially adenocarcinoma, is extremely rare. In these cases, prostatic carcinoma, lung cancer, and colorectum have been described to metastasize to the urethra [3,4]. However, not much information on urethral metastases from these primaries is available so far. To our knowledge, only 9 previous cases with metastases from colorectal cancer have been reported to date [2,5-10]. We report a case of urethral metastasis from a sigmoid colon carcinoma with remaining free of tumor for 34 months, mainly from immunohistopathological point of view to add to some knowledge about its management and mechanism for metastasis.

## Case presentation

A 68-year-old man presented gross hematuria with pain and was hospitalized in June 2011. Four years ago, he had undergone laparoscopic sigmoidectomy for sigmoid colon carcinoma (stage B of Dukes’ classification). Histological examination of the primary tumor showed well to moderately differentiated adenocarcinoma. Postoperative adjuvant chemotherapy was not carried out and he went to hospital regularly for postoperative observation.

**Figure 1:**
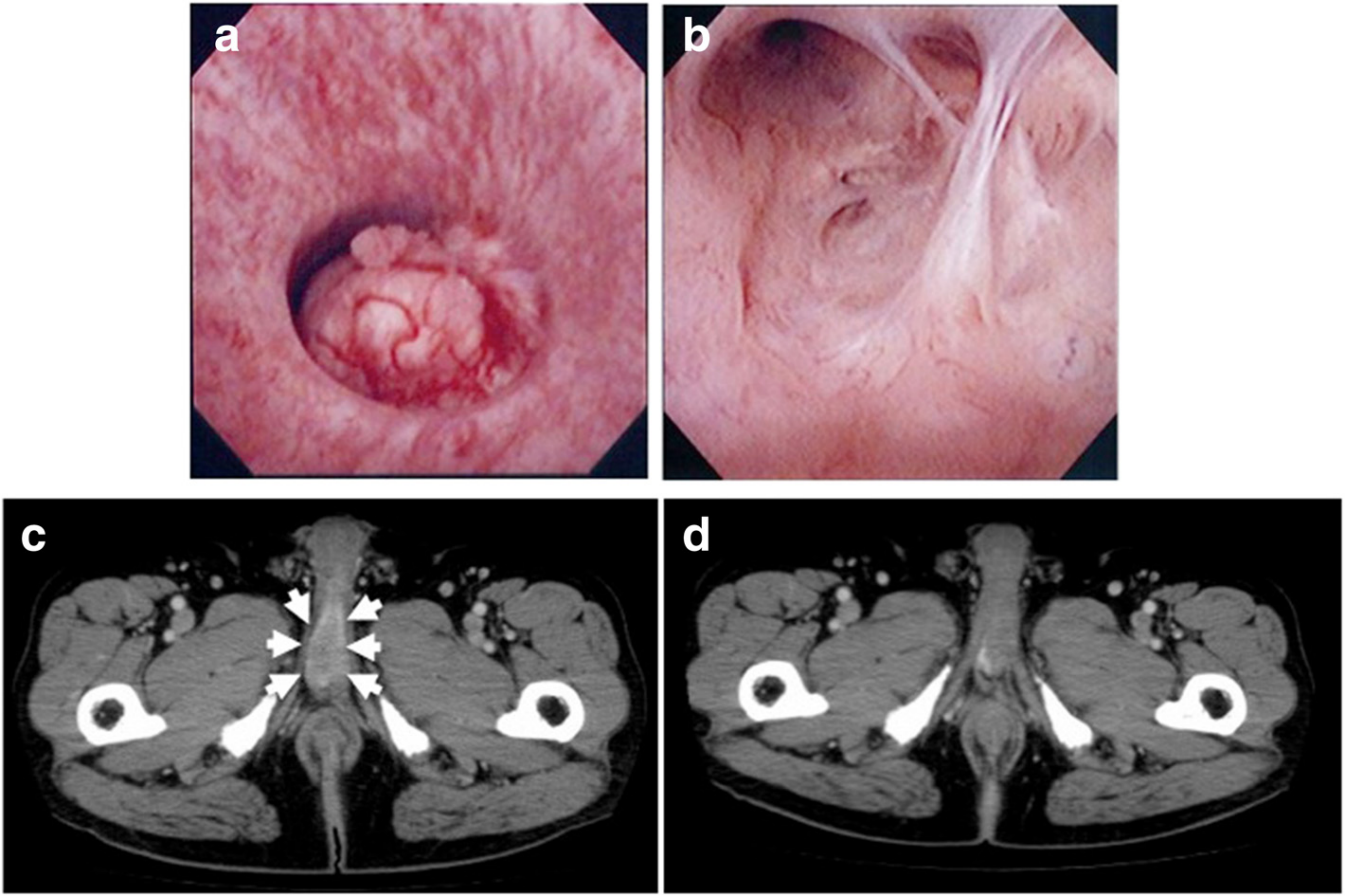
**Urethral tumor detected four years after sigmoidectomy. (a)** Cystoscopy demonstrated papillary tumor of approximate 7 mm in the urethral wall of the distal-potion from the urethral sphincter. **(b)** Cystoscopy demonstrated scar of the transurethral resection without recurrence of the tumor. **(c)** CT-scan imaging showed the small mass of bulbous portion of urethra (white arrow). **(d)** CT-scan imaging showed the total disappearance of urethral metastasis.

**Figure 2:**
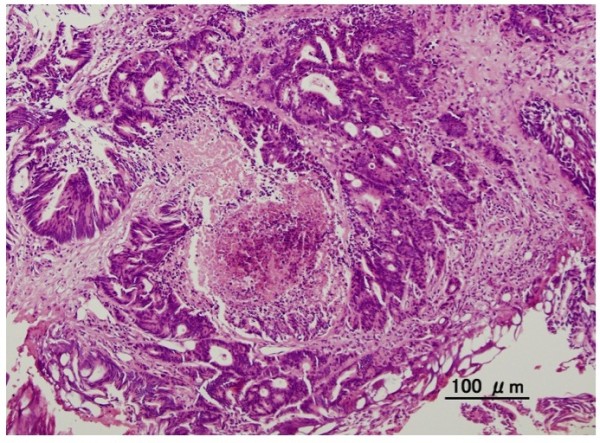
Urethral tumor showed moderately differentiated adenocarcinoma consistent with sigmoid colon cancer (original magnification, ×20).

**Figure 3:**
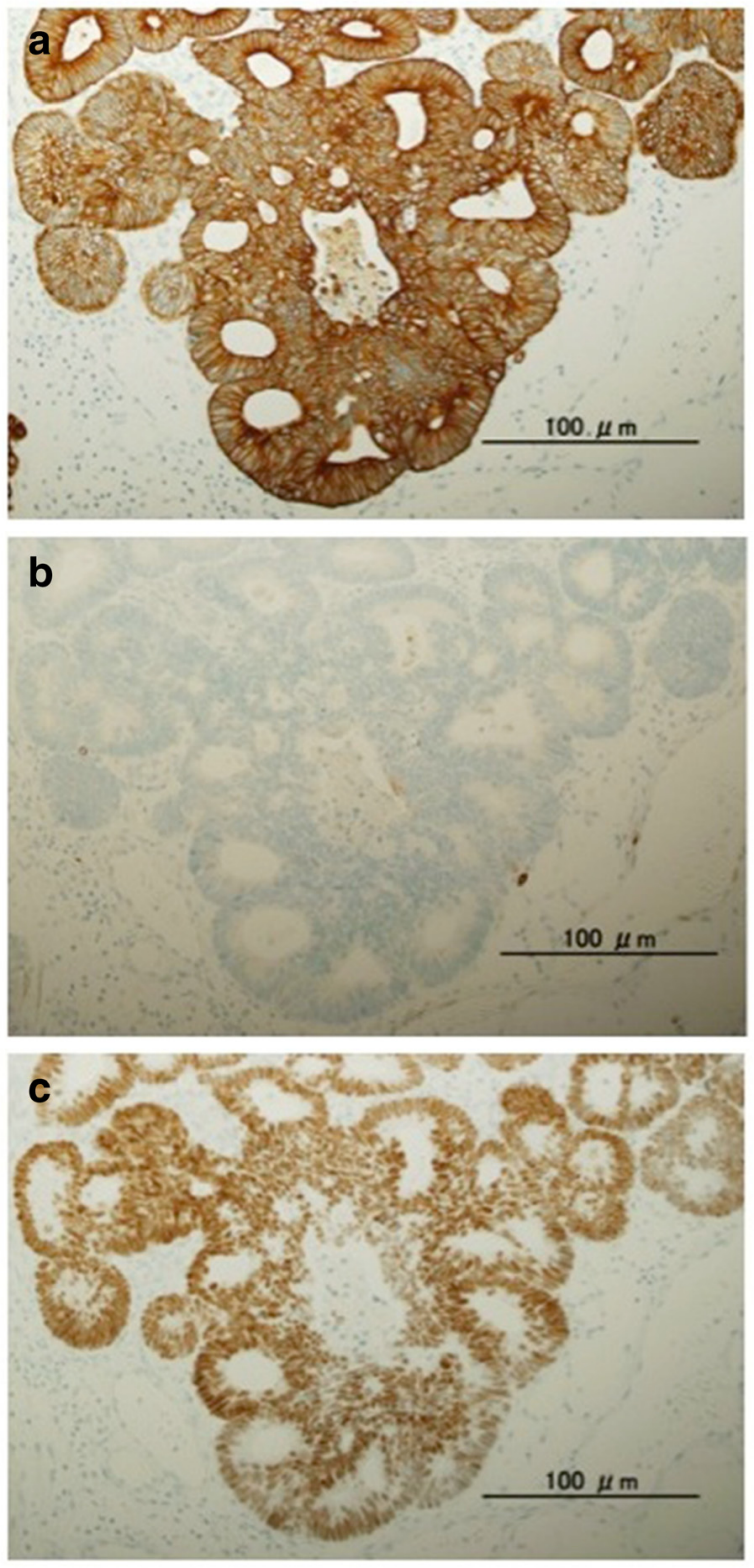
**Immunohistochemical staining of urethral tumor using an anticytokeratin antibody, (a) CK20 and (b) CK7 (original magnification, ×20), and using an antibody against the intestinal epithelia-specific nuclear transcription factor, (c) CDX2 (original magnification, ×20).** The immunohistochemical phenotype showed CK20+/CK7-/CDX2 +.

At the time of hospitalization, nodular induration was not noted. Tumor marker such as the carcinoembryonic antigen was slightly elevated (5.1 ng/mL, normal range, 0–5.0). Urethroscopy identified a papillo-nodular tumor 7 mm in diameter in the bulbar urethra (Figure 1a). Biopsy of the tumor revealed adenocarcinoma, which suggested primary urethral tumor or metastasis of the sigmoid colon cancer. Barium enema examination and colonofiberscopy showed no reccurence of cancer. CT-scan imaging revealed the small mass of bulbous portion of urethra (Figure 1b) and solitary lung metastasis. A transurethral resection of the tumor was performed under the spinal anesthesia. Histological examination showed moderately differentiated adenocarcinoma, which was diagnosed as a metastasis of a sigmoid colon carcinoma pathologically by morphological examination with hematoxylin and eosin staining (Figure 2). Moreover, immunohistochemistry of the urethral tumor showed the positive for cytokertin (CK) 20 and CDX2, the intestinal epithelia-specific nuclear transcription factor, whereas negative for CK7 (Figure 3). These features were consistent with metastatic adenocarcinoma of the sigmoid colon cancer. Additional therapy whether surgical resection (partial penectomy and partial excision of the lung) or systemic chemotherapy was proposed to the patient, and systemic chemotherapy was selected. The patient received 6 cycle of chemotherapy with fluorouracil with leucovorin plus oxaliplatin (FOLFOX4). CT-scan and MRI imaging after 6-cycle FOLFOX4 revealed the total disappearance of both urethral and lung metastasis (Figure 1c). After the induction chemotherapy, the patient received daily tegafur, gimeracil and oteracil (TS-1; 80 mg/day) and was under careful surveillance with CT scan and periodical measurement of serum carcinoembryonic antigen level. Although he is a high-risk patient of recurrent, he is healthy with no evidence of recurrence at about 34 months of follow-up to date.

## Conclusions

We had an experience with the case of urethral metastasis from a sigmoid colon cancer in male. In this case, urethral tumor was considered to have metastasized from colon cancer pathologically by both morphological examination with hematoxylin and eosin staining and immunohistochemical examination. As to the immunohistochemical analysis, the expression of CK20, CK7, and CDX2 was useful for identifying the primary site of metastatic adenocarcinoma. T. Tot summarized the results of 29 studies about for CK20/CK7 phenotype, and stated that colorectal carcinomas showed the CK20+/CK7- phenotype in 78% of the cases and were concluded to be usually CK20+ and CK7- [11]. Therefore, the CK20+/CK7- phenotype indicates metastatic adenocarcinoma, most often from the colorectum. In regard to CDX2, Barbareschi et al. showed that CDX2 immunostained all colorectal adenocarcinomas metastatic to the lung, although it was completely absent in all primary lung neoplasm and in all other adenocarcinoma metastatic to the lung [12]. However, CDX2 is not entirely specific for colorectal cancer, because Weling RW et al. reported that the expression of CDX2 was found ovarian mucinous carcinoma, adenocarcinoma of the urinary bladder, and prostatic adenocarcinoma [13]. The present case exactly showed the phenotype of CK20+/CK7-/CDX2+, suggesting that the urethral tumor was derived from primary tumor of sigmoid colon.

Colorectal cancer with urethral metastasis is generally considered a systemic disease, and the prognosis is generally poor according to the previous case report [8]. Moreover, patients with metachronous metastases were considered to have a worse prognosis than those with synchronous metastases [8]. The clinicopathological features of these 9 patients with urethral metastasis from colorectal cancer are shown in Table 1. The average size of urethral tumor was 2.3 cm. Moreover, the average duration of the detection of metachronous urethral metastasis postoperatively was 2.7 years. In comparison, the urethral metastasis in the present case was detected as a mass with relatively small size and at a long postoperative interval, which might be the reason of the better outcome in current cases. The cases with synchronous reccurence were three, and all cases were female. Among them, two cases (67%) were alive without symptoms of recurrence for 6 and 30 months, respectively. In contrast, the patients with metachronous were 6, and all cases were male including our case. Only two cases (33%) were alive without symptoms of recurrence for 20 and 84 months, respectively. In the present case, both urethral and solitary lung metastasis were detected after curative surgery for sigmoid colon cancer. Additional therapy whether surgical resection or systemic chemotherapy was considered. Recently, neoadjuvant therapy using FOLFOX (with approved biological agent) chemotherapy was recommended as a nonsurgical management of patients with no obstructing metastatic (stage IV) colorectal cancer, and demonstrated excellent result with few complications [14]. This suggests nonsurgical chemotherapy using FOLFOX plus biological antibodies might have beneficial effect on patients with urethral metastasis from colorectal cancer. Therefore, 6-cycle FOLFOX4 was administered to the patient after informed consent about additional therapy, and these metastases were disappeared with no recurrence of disease. This aggressive chemotherapy might help to improve the disease free survival and patient’s overall quality of life.

**Table 1:** Characteristics of the patients with urethral metastasis from colorectal cancer

	**Author**	**Age**	**Gender**	**Primary site**	**Stage**	**Recc**	**Interval**	**Size**	**Symptom**	**Operation**	**Aduvant therapy**	**Outcome**
1	Selikowitz SM et al [5].	48	M	Rectum	NS	M	5Y	NS	Urinary obstruction	None	None	6 M Dead
2		75	M	Sigmoid	Dukes’ D	M	6 M	NS	Slowig of the urinary stream	None	Chemo + iridium	2 M Dead
3	Okaneya T et al [6].	47	M	Sigmoid	Dukes’ C	M	2Y	NS	Gross hematuria	Resection	None	84 M Alive
4	Stragier J et al [7].	68	F	Rectosigmoid	Dukes’ D	S	NS	1 cm	Obstructive micturition	Anterior resection + wedge resection	Rad + Chemo	6 M Alive
5	Kupfer HW et al [8].	67	M	Rectum	Dukes’ B	M	3Y	NS	Voiding difficulties apalpable painless tumor	Partial resection	Rad	10 M Dead
6	Chitale Sv et al [2].	60	F	Sigmoid	Dukes’ B	S	NS	2.5 cm	None	Cystourethrectomy + bil. salphingo oopharectomy	None	30 M Alive
7		72	M	Rectum	Dukes’ B	M	2Y	2 cm	Strangury mild irritative lower urinary tract symptoms	None	None	6 M Dead
8	Chang YH et al [9].	62	M	Ascending	Dukes’ B	M	2Y7M	3.5 cm	Intermittent gross hamaturia	Partial penectomy	Rad + Chemo	20 M Alive
9	Noorani S et al [10].	69	F	Sigmoid	NS	S	NS	NS	Swelling at the urethral opening	Anterior resection + pelvic exentration	None	NS

NS: Not stated; Rec: Recurrence: M: metachronous; S: synchronous; Rad: Radiation; Chemo: Chemotherapy.

Possible mechanisms for metastatic spread to the penis have been described as direct arterial extension, secondary and tertiary embolism, instrumental spread, paradoxical embolism, retrograde lymphatic spread and direct extension [15]. Of these, the latter three mechanisms are considered to be most likely, when the primary tumor arises from the rectosigmoid colon. Batson OV described that the communication between pelvic and vertebral veins would easily account for retrograde venous spread during Valsalva maneuvers when proximal venous channels are blocked with tumor [16]. Selikowitz SM et al. also described that blockage of proximal lymphatics might allow retrograde lymphatic spread to occur via connections between inferior hemorrhoidal and pudendal lymphatics, and direct extension was possible from the ischiorectal fossa, through the junction of Collees fascia with the triangular ligament, to the superficial perineal pouch. Nevertheless, the number of the cases with urethral metastasis was few. Moreover, only 3 cases (33%) out of nine cases that were reported previously had lymph node metastasis (stage C of Dukes’ classification) or distant metastasis (stage D of Dukes’ classification). The accumulation of these cases is necessary for exact clarification of the mechanism for metastasis to the urethra.

In patients with colorectal cancer, postoperative follow-up, including tumor markers (carcinoembryonic antigen, carbohydrate antigen 19–9, and serum p53 antibody), chest X-ray, liver ultrasound, computed tomography, and colonofiberscopy is routinely performed. Urethral metastasis from colorectal cancer is a very rare occurrence. Therefore, the examination of the urinary system as a part of the routine postoperative follow-up protocol would not be justified. However, in the presence of urinary symptoms, the possibility of the urethral metastasis should be considered.

### Consent

Written informed consent was obtained from the patient for publicatin of this Case report and any accompanying images. A copy of the written consent is available for review by the Editor of this journal.

## Abbreviations

SEER: Surveillance, epidemiology and end results; CK: Cytokertin; FOLFOX: Fluorouracil with leucovorin plus oxaliplatin; TS-1: Tegafur, gimeracil and oteracil.

## Competing interests

The authors declare that they have no competing interests. No financial support has been received.

## Authors’ contributions

SK drafted the manuscript. And conducted a literature search. JK, ES and TW conducted a literature search and contributed to drafting the manuscript. AN, AN and YH performed the operation and reviewed the manuscript and gave final approval for publication. All authors read and approved the final manuscript.

## Pre-publication history

The pre-publication history for this paper can be accessed here:

http://www.biomedcentral.com/1471-2482/14/31/prepub
